# Patchouli Alcohol Improves the Integrity of the Blood-Spinal Cord Barrier by Inhibiting Endoplasmic Reticulum Stress Through the Akt/CHOP/Caspase-3 Pathway Following Spinal Cord Injury

**DOI:** 10.3389/fcell.2021.693533

**Published:** 2021-07-22

**Authors:** Chongan Huang, Weiqi Zhang, FeiFan Chu, Hao Qian, Yining Wang, Fangzhou Qi, Mengke Ye, Jiaying Zhou, Zhi Lin, ChenLin Dong, Xiangyang Wang, Qingqing Wang, Haiming Jin

**Affiliations:** ^1^Department of Orthopaedics, The Second Affiliated Hospital and Yuying Children’s Hospital of Wenzhou Medical University, Wenzhou, China; ^2^The First Affiliated Hospital, Wenzhou Medical University, Wenzhou, China

**Keywords:** central nervous system, spinal cord injury, blood-spinal cord barrier, patchouli alcohol, endoplasmic reticulum stress

## Abstract

Spinal cord injury (SCI) is a destructive and complex disorder of the central nervous system (CNS) for which there is no clinical treatment. Blood-spinal cord barrier (BSCB) rupture is a critical event in SCI that aggravates nerve injury. Therefore, maintaining the integrity of the BSCB may be a potential method to treat SCI. Here, we showed that patchouli alcohol (PA) exerts protective effects against SCI. We discovered that PA significantly prevented hyperpermeability of the BSCB by reducing the loss of tight junctions (TJs) and endothelial cells. PA also suppressed endoplasmic reticulum stress and apoptosis *in vitro*. Furthermore, in a rat model of SCI, PA effectively improved neurological deficits. Overall, these results prove that PA exerts neuroprotective effects by maintaining BSCB integrity and thus be a promising candidate for SCI treatment.

## Introduction

Spinal cord injury (SCI) is a disorder associated with high mortality that not only leads to physical disabilities but also imposes a large economic burden (Schwab et al., 2018; Badhiwala et al., 2019; James et al., 2019). Neurological impairment associated with SCI can be classified into two phases: primary acute injury and subsequent secondary injury (Ahuja et al., 2017a, b). Secondary injury dramatically aggravates neuronal death and expands the damaged area (Tran et al., 2018). Because of the irreversibility of mechanical injury, therapeutic strategies for SCI mainly alleviate secondary injury.

The pathophysiological process of secondary nerve injury is mostly related to blood-spinal cord barrier (BSCB) rupture (Lee et al., 2016). The BSCB comprises non-fenestrated endothelial cells and perivascular cells interconnected by tight junctions (TJs). The BSCB is vital to maintain microenvironment homeostasis and control material exchange (Sauer et al., 2017). Post-SCI, the permeability of the BSCB to immune cells and neurotoxic substances is increased, leading to neuronal and glial cell apoptosis and irreversible neurological disability (Bartanusz et al., 2011; Kumar et al., 2017). Thus, preventing BSCB rupture is considered a potential strategy for SCI treatment.

Oxidative stress is important for BSCB disruption in various pathological conditions (Zhou et al., 2016a; He et al., 2017). Under stressful conditions, ER stress, or disruption of the function of the endoplasmic reticulum (ER) in cells, is induced. Following exposure to excessive stress, prolonged ER stress activates the ER-associated cell death pathway (Schröder, 2008; Oakes and Papa, 2015). Previous studies have indicated that BSCB disruption is directly related to ER stress-mediated apoptosis (Zhou et al., 2017) and that the transcriptional activation of the protein CHOP, which is homologous to CCAAT enhancer-binding protein (C/EBP), is considered an apoptotic pathway (Hu et al., 2018). Evidence has indicated that GRP78/CHOP triggers apoptosis pathways that cause cell death under oxidative stress conditions (Ayaub et al., 2016; Zhao et al., 2018).

Recently, studies have also confirmed that mitochondrial damage results from ER stress and is coupled with ER stress-mediated apoptosis (Kim et al., 2019). Mitochondrial damage is considered a crucial component of the pathogenesis of endothelial cell death in animal models of SCI. Studies have shown that overexpression of oxidative stress-induced Bcl-2 family proteins results in mitochondrial damage, leading to apoptosis (Shore et al., 2011; Iurlaro and Muñoz-Pinedo, 2016). The Bcl-2 family of proteins contains multiple members that regulate mitochondrial integrity, apoptosis, and other cellular processes (Siddiqui et al., 2015). The Bcl-2 family promotes the activation of caspase-9, which then activates caspase-3 and caspase-7 to elicit the mitochondrial pathway of apoptosis (Youle and Strasser, 2008).

Patchouli alcohol (PA, C15H26O) is a natural tricyclic sesquiterpene isolated from patchouli that has traditionally been used to treat infectious diseases. Previous studies have shown that PA has anti-influenza (Wu et al., 2011), antitumorigenic (Jeong et al., 2013), anti-inflammatory, and neuroprotective activities (Wei et al., 2018). However, the impact of PA on SCI remains unclear. Here, we demonstrate that PA may protect the integrity of the BSCB by inhibiting ER stress and reducing mitochondrial damage after SCI.

## Materials and Methods

### Reagents and Antibodies

Patchouli alcohol was provided by Chengdu Herbpurify Co., Ltd., and the purity was 98%. Endothelial cell medium (ECM) and fetal bovine serum (FBS) were provided by Invitrogen (Carlsbad, CA, United States). Anti-Akt, anti-p-Akt (Ser473), anti-cleaved-caspase-3, anti-cleaved-caspase-9, anti-Bax, anti-Bcl-2, anti-CHOP, anti-glucose-regulated protein (GRP-78), anti-ATF-6, and anti-GAPDH antibodies were provided by Santa Cruz Biotechnology (Santa Cruz, CA, United States). HRP-conjugated goat anti-rabbit and anti-mouse IgG were provided by Cell Signaling Technology Inc. (Danvers, MA, United States). An enhanced chemiluminescence (ECL) kit was provided by Bio-Rad (Hercules, CA, United States). LY294002 (a PI3K/Akt inhibitor) and all other reagents were provided by Sigma.

### Cell Culture

Human brain microvascular endothelial cells (HBMECs) were obtained from a commercial source (ScienCell, Carlsbad, CA, United States) and cultured in ECM. Approximately 1 × 10^6^ cells in 5 ml of medium were added to each Petri dish and subsequently placed in an incubator (37°C, 5% CO_2_ and 95% air). The cells spread on the bottom of the plate after 24 h and reached confluence in 3–4 days. To simulate spinal cord injury *in vitro*, HBMECs were exposed to oxygen-glucose deprivation (OGD). The medium was changed to sugar-free basic medium. The cells were then cultured in 60-mm or 35-mm Petri dishes coated with poly-D-lysine and starved overnight in 0.5% FBS. Next, HBMECs were cultured in hypoxic chambers (1029; Thermo Fisher Scientific, Waltham, MA, United States) for 12 h (oxygen concentration <0.2%). Subsequently, the cell medium was replaced with ECM, and PA (2.5, 5, 10, or 20 μmol/L) was added.

### Drug Screening Assay

To determine whether PA can maintain the integrity of HBMECs, HBMECs were pretreated with natural compounds (10 μM for the drug screening assays) and varying concentrations of PA. Endothelial monolayer permeability was evaluated based on the intensity of FITC-dextran (Natarajan et al., 2018). HBMECs were placed on the upper side of the insert and allowed to aggregate. FITC-dextran (1 mg/ml) was applied to the endothelial monolayer after drug exposure for 2 h. Next, the plate was read using a fluorescence plate reader at 493 nm (excitation wavelength) and 517 nm (emission wavelength) (SpectraMax M2e; Molecular Devices, Sunnyvale, CA, United States) to determine the relative fluorescence that passed through the chamber.

### Animal Model and Drug Treatment

Sixty female Sprague-Dawley rats weighing between 200 and 220 g were obtained from the Animal Center of the Chinese Academy of Sciences (Shanghai, China) and bred in a specific pathogen-free (SPF) laboratory. After 1 week of adaptation, the rats were anesthetized with 2% (w/v) pentobarbital sodium (2 mL/kg, i.p.). Rats in the sham group endured the exposure of spinal cords while other rats were subjected to SCI. Then a laminectomy was performed at the T9 vertebra. For SCI rats, when the spinal cord was fully exposed, a MASCIS Impactor with a 2.5-mm diameter tip and a 10-g rod with a drop height of 12.5 mm (W.M. Keck Center for Collaborative Neuroscience at Rutgers, The State University of New Jersey) was used to induce contusion SCI in rats. Post-operatively, carprofen (5 mg/kg s.c.) was used to relieve the pain of the rats and the rats were randomly divided into three groups (sham, SCI, and SCI + 10 mg/kg PA). PA was suspended in 0.1% Tween 80 in phosphate-buffered saline (PBS) and administered intraperitoneally at a dosage of 10 mg/kg/day for consecutive 56 days. Rats from the sham and SCI groups received an equal volume of 0.1% Tween 80 instead. The rats were housed following an injury after the muscles and skin were closed in layers, and the bladder was emptied manually twice a day. All the animal experiments were conducted in accordance with the ethics committee of the National Institutes of Health, China, and performed according to the National Institutes of Health Guide for Care and Use of Laboratory Animals (NIH Publications No. 8023, revised 1978).

### BSCB Permeability Measurement

Blood-spinal cord barrier maintenance was evaluated based on the extravasation of Evans blue dye (Zheng et al., 2016). One day after SCI, 2% Evans blue dye (4 mL/kg; Sigma) was administered through the tail vein of each rat. The dye was allowed to circulate for 2 h, and then the rats were anesthetized, sacrificed, and perfused with 0.9% saline via the left cardiac ventricle. The spinal cords were then removed. Some of the tissues were weighed and soaked in N, N0-dimethylformamide (Jin San, Wenzhou, China) for 72 h (50°C) and then centrifuged. The optical density of the supernatant was detected at 620–680 nm using an enzyme-labeled meter. The amount of dye in the samples was normalized to the mass (μg/g) of spinal cord tissue using a standardized curve. The remaining spinal cord tissues were fixed by perfusion with 4% paraformaldehyde and sectioned into 30-μM coronal slices with a cryostat. A confocal fluorescence microscope was employed to observe Evans blue staining.

### Behavioral Assessments

The Basso, Beattie, and Bresnahan (BBB) locomotor rating scale was used to assess the locomotor ability of the animals on a 22-point (0–21) scale assessing hindlimb locomotor function and open-field locomotion (Lee et al., 2017). Before injury and 3, 7, 14, 21, 28, and 56 days after injury, the rats were placed in an open-field chamber. Animals showing more normal locomotion were assigned a higher score. The inclined plane test was applied to evaluate the sensorimotor function of the rats (Hayashi et al., 2019). The rats were placed on a tilting board, and the tilt angle was gradually increased. The maximum angle at which each rat could stay on the board for 5 s without sliding was recorded, and the average value was obtained after 3 trials. The behavioral tests were performed by two independent observers in a double-blind manner.

### Tissue Collection

The animals were divided into three groups: sham, SCI, and SCI + PA. At specific time points after SCI, an equal number of rats was selected from each group, sacrificed by exposure to excessive CO_2_ and initially perfused with 0.1 M phosphate-buffered saline (PBS) via cardiac puncture. For hematoxylin and eosin staining, immunohistochemistry, the spinal cords were removed, post-fixed in 4% paraformaldehyde overnight, and subsequently embedded in paraffin. The paraffin-embedded sections (5 μm thick) were mounted on slides for the subsequent step. For molecular studies, the spinal cord segment at the injury site was immediately isolated and frozen at −80°C.

### Reverse Transcription-Polymerase Chain Reaction (RT-PCR)

After the cells were grouped and processed, total RNA was extracted using TRIzol reagent and then reverse transcribed into cDNA. Next, the samples were denatured at 94°C for 10 min and amplified at 94°C for 15 s, 60°C for 60 s, and 72°C for 60 s for 40 cycles. The primer sequences were as follows: ZO-1 sense: 5′-AGGTGAAACTCTGCTGAGCC-3′; ZO-1 antisense: 5′-TCCAGTGTGGCAAG CGTAG −3′; Claudin-5 sense: 5′-GAACTACGTCTAAGGGCGGG-3′; Claudin-5 antisense: 5′-AATTCAGCGGTG GTCGTCAT −3′; GRP78 sense: 5′-GTGCCCACCAAGAAGTCTCA −3′; and GRP78 antisense: 5′-TTTTGTCAGG GGTCGTTCACC −3′. An RT-PCR system (ABI) was used for quantification, and all the reactions were performed three times.

### Immunofluorescence and Immunohistochemistry

To measure the necrosis or death of motor neurons, paraffin-embedded spinal cord sections were stained with hematoxylin and eosin according to the manufacturer’s instructions. For immunofluorescence, when the cells were approximately 70% confluent, they were fixed with 4% paraformaldehyde for 15 min at room temperature. Next, the cells were washed three times in PBS for 5 min. For immunohistochemistry, spinal cord sections were incubated in citric acid buffer (pH 6.0) in a microwave for antigen retrieval and cultured at room temperature for 1 h in blocking buffer. The blocking buffer comprised 5% bovine serum albumin (BSA) in PBS-0.1% Triton X-100 (Sigma). The slides were incubated at 4°C overnight with the following primary antibodies: ZO-1 (1 μg/ml, Abcam), β-Catenin (1:250, Abcam), and Claudin5 (1:200, Santa Cruz). Following primary antibody incubation, the slides were incubated with secondary antibody, and the cells were then incubated with the nuclear marker Hoechst 33258 (1 μg/ml; Sigma, Oakville, Ontario, Canada). All the images were captured using a confocal fluorescence microscope (Nikon, Japan).

### Western Blot Analysis

Protein extraction reagents were used to extract protein from spinal cord tissues and HBMECs. All the proteins from the tissues and cells were separated by SDS-PAGE and transferred to PVDF membranes. The membranes were incubated with the following primary antibodies overnight at 4°C for analysis: ZO-1 (1 μg/ml, Abcam), β-Catenin (1:250, Abcam), and Claudin5 (1:200, Santa Cruz, Jerusalem, Israel). GAPDH (1:10,000; Sigma) was used as a loading control. Alexa Fluor 568-, Alexa Fluor 488-, or Alexa Fluor 647-conjugated donkey anti-rabbit or anti-mouse secondary antibodies were used. The signals were visualized using a ChemiDocXRS + Imaging System (Bio-Rad) for statistical analysis. All the experiments were repeated three times.

### Cell Viability Determination

Cell Counting Kit 8 (CCK-8) was used to measure the toxic effect of PA on HBMEC proliferation according to the manufacturer’s instructions. HBMECs were induced to proliferate and centrifuged to generate a cell suspension. The PA groups were treated with 1, 2.5, 5, 10, 20, 50, or 100 μmol/L PA, and the untreated group served as a control. Each group of cells was then seeded in 96-well plates at a density of 1 × 10^5^ cells/well and allowed to proliferate for 48 h. Next, 10 μL of CCK-8 solution was added to each well, and the cells were incubated for 3 h in a 37°C incubator at 5% CO_2_. Finally, the plate was placed in a microplate reader, and the absorbance was measured at 450 nm. The experiment was repeated three times.

### TUNEL Assay

The One-Step TUNEL Apoptosis Assay Kit (Roche, Mannheim, Germany) was used to evaluate DNA fragmentation *in vivo*. Images were taken using a Nikon ECLIPSE Ti microscope (Nikon, Melville, NY, United States). The apoptosis ratio of HBMECs was determined using a PI/Annexin V-FITC kit (Invitrogen). Next, a FACScan flow cytometer (Becton Dickinson, Franklin Lakes, NJ, United States) was employed for analysis according to the kit’s manual.

### JC-1 Fluorescence Assay for MMP Measurement

Mitochondrial JC-1 fluorescence was measured to determine the mitochondrial membrane potential (MMP) (Chazotte, 2011). At low mitochondrial polarization (MMP < 100 mV), JC-1 remains a monomer and emits green fluorescence in the fluorescein isothiocyanate (FITC) channel; at high mitochondrial polarization (MMP > 140 mV), JC-1 forms J-aggregates and emits red fluorescence (Yang et al., 2019a). HBMECs were cultured with JC-1 solution at 37°C for 20 min. The cells were then washed twice with JC-1 buffer, and medium was added to each well. Images were captured using a fluorescence microscope (Olympus, Tokyo, Japan). The MMP was determined by the ratio of red to green fluorescence.

### Statistical Analysis

All the data were expressed as means ± standard error of the mean (SEM). Either two-tailed Student’s *t*-test or one-way analysis of variance (ANOVA) and Dunnett’s *post hoc* test were implemented for pairwise comparisons between all the experimental groups unless otherwise stated. *P* < 0.05 was considered statistically significant.

## Results

### PA Protects OGD-Exposed HBMECs

**FIGURE 1:**
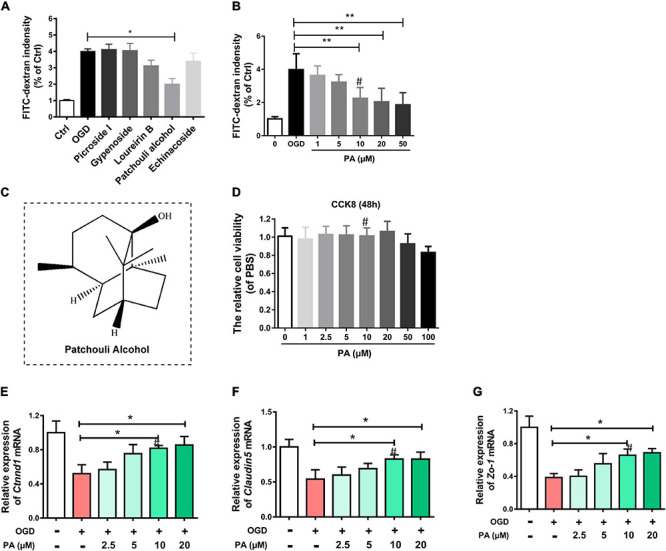
PA protects OGD-exposed HBMECs. **(A,B)** FITC-dextran assay following drug treatment. **(C)** Molecular structure of PA. **(D)** The CCK-8 assay was used to evaluate the effect of various concentrations of PA on the proliferation of HBMECs. **(E–G)** Expression of Ctnnd1, Claudin-5, and Zo-1 under different conditions, as detected by RT-PCR. The expression of Ctnnd1, Claudin-5, and Zo-1 was increased following treatment at doses of 10∼20 μmol/L. “**” represents *P* < 0.01 and “*” represents *P* < 0.05. All the experiments were repeated three times. The data are presented as means ± SD; *n* = 3. ^``#”^ represents the optimal concentration we choose.

Several drugs that may have therapeutic effects on OGD-exposed HBMECs were screened. The FITC-dextran assay showed that, compared with other drugs, PA had a more significant effect on decreasing the permeability of HBMECs in a dose-dependent manner (Figures 1A–C). The CCK-8 assay was performed to assess the cytotoxicity of PA and determine the appropriate concentration. The test results (Figure 1D) indicated that, compared with the control treatment (0 μmol/L PA), PA had no detectable effect on the proliferation of HBMECs in this concentration range (0–100 μmol/L). TJ proteins and adherens junction (AJ) proteins are critical to maintain TJs in the BSCB. HBMECs were cultured *in vitro*, and OGD was used to mimic the microenvironment after SCI. The RT-PCR results are shown in Figures 1E–G. PA increased the expression levels of TJ proteins and AJ proteins in a dose-dependent manner. At 10 μmol/L, the effect was close to the optimal effect (^∗^*P* < 0.05).

### PA Treatment Increases AJ and TJ Protein Expression in HBMECs

**FIGURE 2:**
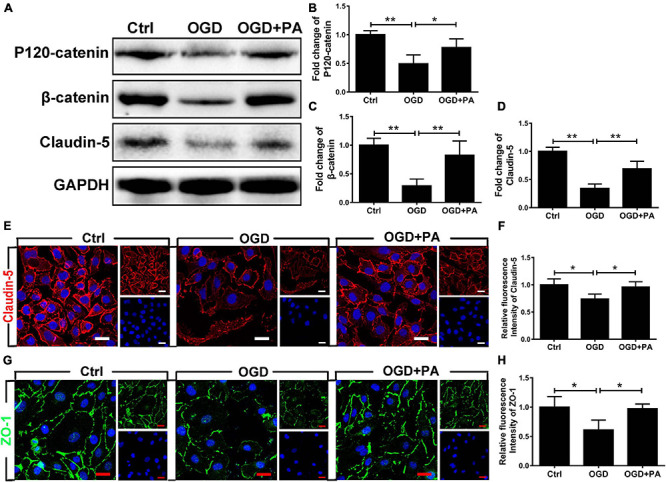
PA treatment increases the expression of AJ and TJ proteins. **(A)** Representative Western blots of P120-catenin, β-catenin, and claudin-5 expression in HBMECs from each group, with GAPDH as the protein loading control. **(B–D)** Quantification of the protein expression data for each group in **(A)**. **(E–G)** Representative images of immunofluorescence staining for Claudin-5 (red) and Zo-1 (green) around HBMECs in each group of cells (original magnification, 200×). The nuclei are labeled with DAPI (blue) for each group of cells. **(F–H)** Quantification of immunofluorescence staining. “**” represents *P* < 0.01 and “*” represents *P* < 0.05. The data are presented as means ± SD; *n* = 3.

Tight junctions and AJs are vital parts of the BSCB according to previous reports (Liu et al., 2014). To confirm the effect of PA on HBMECs, we used OGD to treat. The Western blot results are presented in Figure 2A. The expressions of claudin-5, β-catenin, and P120-catenin in the OGD treatment group were notably lower than those in the control group (Figures 2B–D; ^∗∗^*P* < 0.01). The expression of claudin-5, β-catenin, and P120-catenin was significantly increased in the PA-treated group compared with that in the OGD-exposed group (Figures 2B–D; ^∗^*P* < 0.05; ^∗∗^*P* < 0.01). We also performed immunofluorescence to more intuitively visualize the changes in protein expression. The images (Figures 2E–G) showed that PA treatment significantly reversed the decrease in Claudin-5 and ZO-1 protein expression around HBMECs induced by OGD exposure (Figures 2F–H; ^∗∗^ *P* < 0.01, ^∗^*P* < 0.05). The above results indicated that PA effectively reduces the loss of TJ and AJ proteins induced by OGD, thereby reducing the permeability of the BSCB.

### PA Suppresses the Activation of ER Stress Caused by OGD

**FIGURE 3:**
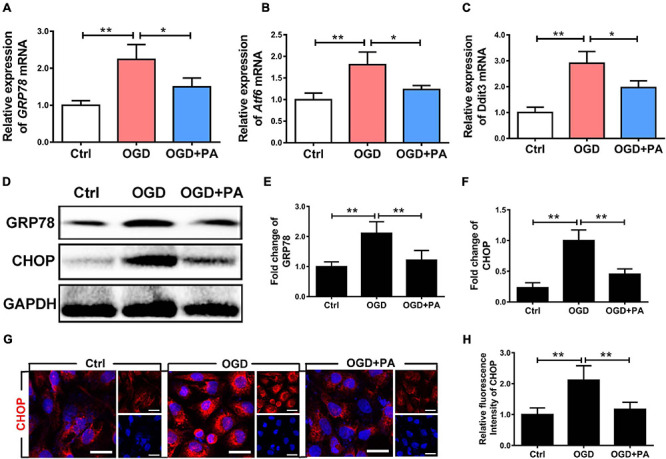
PA suppresses the activation of ER stress caused by OGD. **(A–C)** Expression of GRP78, Atf6, and Ddit3 in cells under different conditions, as detected by RT-PCR. **(D)** Representative Western blots of GRP78 and CHOP expression in HBMECs from the control group, OGD group and PA group, with GAPDH as the protein loading control. **(E,F)** Quantification of Western blot protein expression data in each group. **(G)** Representative images of immunofluorescence staining for CHOP (red) in each group of cells. **(H)** Quantification of immunofluorescence staining. The level of CHOP was significantly reduced in the PA treatment group compared with that in the OGD group. “**” represents *P* < 0.01 and “*” represents *P* < 0.05. The data are presented as means ± SD; *n* = 3.

The essence of the ER stress response is the accumulation of UPs. In the early stage, molecular chaperones such as GRP78 are recruited to allow adaptation to UP accumulation. When decompensated, the transcription factor CHOP is activated to induce apoptosis. The expression of the CHOP gene is positively regulated by transcription factors such as activating transcription factor 6 (ATF6), XBP-1, and ATF4. PA treatment reduced the expression levels of the ER stress-related molecules GRP78, ATF6, and DDIT3 under OGD conditions (Figures 3A–C; ^∗^*P* < 0.05; ^∗∗^*P* < 0.01). Figure 3D shows that, after OGD exposure, the expression levels of CHOP and GRP78 in HBMECs were significantly increased (Figures 3E,F; ^∗^*P* < 0.05; ^∗∗^*P* < 0.01). PA treatment effectively inhibited the excessive activation of early ER stress, suggesting that PA treatment reduces the accumulation of UPs in the ER during ER dysfunction. PA treatment also effectively inhibited the expression of CHOP when the ER was overactivated by stress (Figure 3F; ^∗^*P* < 0.05). The immunofluorescence results showed that PA reduced the expression of CHOP induced by OGD (Figures 3G,H; ^∗∗^*P* < 0.01). The above results indicate that PA reduces the expression levels of ER stress-related proteins by alleviating the early UP response in the ER, reducing the protein expression level of CHOP and reducing the damage resulting from excessive ER activation.

### PA Inhibits Endothelial Apoptosis by Affecting Mitochondrial Function

**FIGURE 4:**
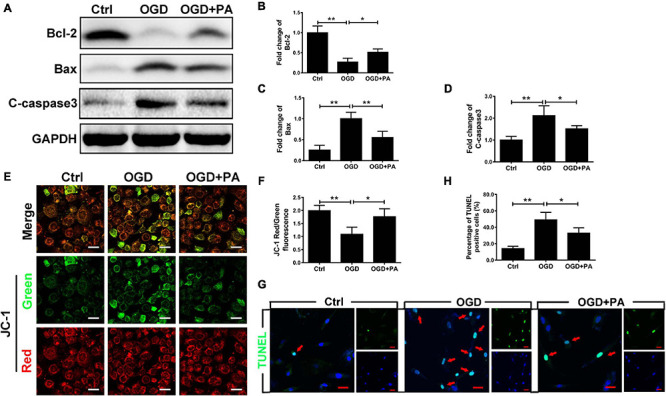
PA inhibits endothelial cell apoptosis by affecting mitochondrial function. **(A)** Representative Western blots of Bcl-2, Bax, and C-casp3 expression in HBMECs from the control, OGD and PA groups, with GAPDH as the protein loading control. **(B–D)** Quantification of Western blot protein expression data in each group. **(E)** Typical images of immunofluorescence staining of JC-1 in each group of cells. **(F)** Quantification of immunofluorescence staining. The level of JC-1 red/green fluorescence was increased in the PA treatment group compared with that in the OGD group. **(G)** Representative images of TUNEL staining in each group of cells. **(H)** Quantification of immunofluorescence staining. The number of viable cells was significantly increased in the PA treatment group compared with that in the OGD group. “**” represents *P* < 0.01 and “*” represents *P* < 0.05.

Endoplasmic reticulum stress induces the death of mitochondria and leads to apoptosis (Kim et al., 2019). The Bcl-2 family is related to apoptosis (Youle and Strasser, 2008). After OGD treatment, the expression of Bax and C-casp3 (proapoptotic proteins) in HBMECs was significantly increased (Figure 4A), while the expression of Bcl-2 (an antiapoptotic protein) was decreased (Figures 4B–D; ^∗^*P* < 0.05; ^∗∗^*P* < 0.01). Additionally, PA effectively increased Bcl-2 expression and inhibited Bax and C-casp3 expression when the ER was overactivated by stress (Figures 4B–D; ^∗^*P* < 0.05; ^∗∗^*P* < 0.01). Furthermore, we applied JC-1 to measure the MMP. Normally, JC-1 forms aggregates that emit strong red fluorescence in the mitochondrial matrix; however, in damaged mitochondria, JC-1 exists in the cytoplasm as a monomer because of the loss of membrane potential and produces green fluorescence. PA reduced OGD-induced mitochondrial death (Figures 4E,F; ^∗^*P* < 0.05; ^∗∗^*P* < 0.01). Additionally, TUNEL staining was used to detect apoptosis in HBMECs (Figures 4G,H; ^∗^*P* < 0.05; ^∗∗^*P* < 0.01). The results proved that PA inhibited endothelial cell apoptosis after OGD treatment. The above results suggested that PA inhibits the loss of mitochondrial function and reduces the expression levels of apoptosis-related proteins, thereby reducing endothelial cell apoptosis.

### PA Reduces Mitochondrial Damage by Inhibiting ER Stress

**FIGURE 5:**
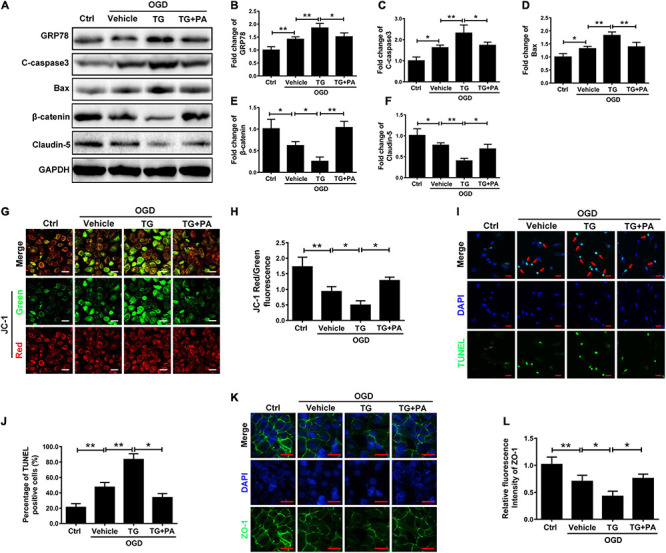
PA reduces mitochondrial damage by inhibiting ER stress. **(A)** Representative Western blots of GRP78, Bax, C-casp3, β-catenin, and Claudin-5 expression in HBMECs from the control, OGD, TG + OGD, and PA + OGD groups, with GAPDH as the protein loading control. **(B–F)** Quantification of Western blot protein expression data in each group. **(G)** Representative images of JC-1 staining in each group of cells. **(H)** Quantification of immunofluorescence staining. The level of JC-1 red/green was markedly decreased in the TG treatment group compared with that in the OGD group, and the level of JC-1 red/green was significantly increased in the PA treatment group compared with that in the TG + OGD group. **(I)** Representative images of TUNEL staining in each group of cells. **(J)** Quantification of immunofluorescence staining. **(K)** Representative images of immunofluorescence staining for Zo-1 (green) around HBMECs in each group (original magnification, 200×). The nuclei are labeled with DAPI (blue) in each group of cells. **(L)** Quantification of immunofluorescence staining. “**” represents *P* < 0.01 and “*” represents *P* < 0.05. The data are presented as means ± SD; *n* = 3.

To verify the relationship between ER stress and mitochondrial damage, the ER stress inducer thapsigargin (TG) was applied. TG increased the expression levels of GRP78, C-caspase3, and Bax and decreased β-catenin and claudin-5 expression, whereas PA treatment reversed the effects of TG (Figures 5A–F). Furthermore, JC-1 immunofluorescence showed that TG significantly induced mitochondrial death (Figures 5G,H; ^∗∗^*P* < 0.01; ^∗^*P* < 0.05). TG induced HBMEC apoptosis after OGD exposure and PA reversed this effect (Figures 5I,J). Additionally, immunofluorescence showed that TG suppressed the expression of Zo-1 (Figures 5K,L; ^∗∗^*P* < 0.01; ^∗^*P* < 0.05). Thus, ER stress induces mitochondrial damage and subsequent cell apoptosis, and PA prevents mitochondrial damage by inhibiting ER stress.

### AKT Is Involved in PA-Mediated ER Stress Inhibition

**FIGURE 6:**
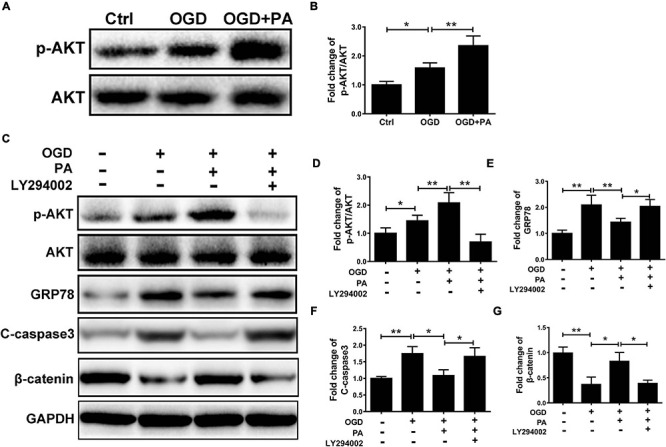
AKT is involved in PA-mediated ER stress inhibition. **(A)** Representative Western blots of p-AKT and AKT expression in HBMECs from the control, OGD and PA + OGD groups. **(B)** Quantification of Western blot p-AKT/AKT ratio data in each group. **(C)** Representative Western blots of p-AKT, AKT, GRP78, C-casp3, and β-catenin expression in HBMECs from the control, OGD, PA + OGD, and LY294002 + PA + OGD groups. **(D)** Quantification of Western blot p-AKT/AKT ratio data in each group. **(E–G)** Quantification of Western blot protein expression data in each group. “**” represents *P* < 0.01 and “*” represents *P* < 0.05.

To investigate whether PA inhibits ER stress activation in endothelial cells in an AKT-dependent manner, HBMECs were treated with LY294002, a classic PI3K inhibitor. The phosphorylation of AKT was increased by PA treatment (Figures 6A,B), and LY294002 markedly suppressed the phosphorylation of AKT in HBMECs co-treated with PA and LY294002 (Figures 6C–G). Furthermore, PA reduced GRP78, C-caspase3, and β-catenin expression by activating AKT; this effect was abolished when PA was combined with LY294002 (a specific inhibitor of PI3K) (Figures 6C–G). The above results showed that AKT is involved in PA-mediated inhibition of ER stress.

### PA Prevents ER-Induced Damage to the BSCB After SCI

**FIGURE 7:**
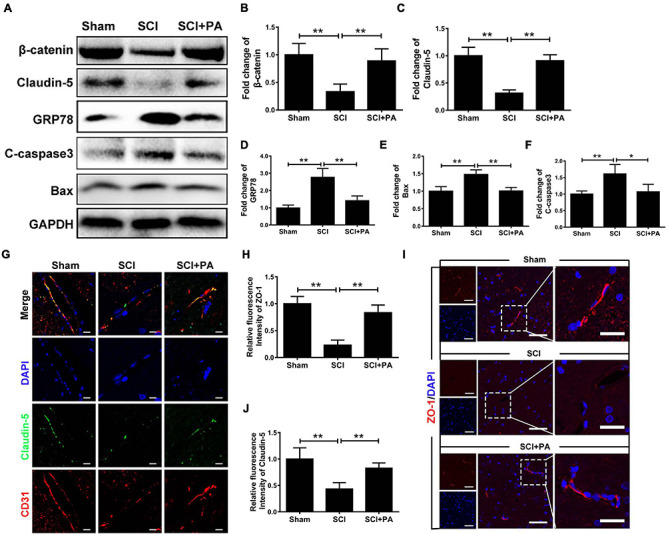
PA prevents ER-induced damage to the BSCB after SCI. **(A)** Representative Western blots of GRP78, β-catenin, Claudin-5, Bax, and C-casp3 expression in control, SCI and PA-treated groups, with GAPDH as the protein loading control. **(B–F)** Quantification of the protein expression data in each group shown in **(A)**. **(G)** Representative images of immunofluorescence staining for Claudin-5 (green) and CD31 (red) in each group. The nuclei are labeled with DAPI (blue) in each group. **(H–J)** Quantification of immunofluorescence staining. **(I)** Representative images of immunofluorescence staining for Claudin-5 (red) in each group. The nuclei are labeled with DAPI (blue) in each group. “**” represents *P* < 0.01 and “*” represents *P* < 0.05. All the experiments were repeated three times. The data are presented as means ± SD; *n* = 8 per group.

To prove that the ER stress level is increased and BSCB is disrupted after SCI, a rat model of SCI was established. The rats were intraperitoneally injected with PA. After 1 day, tissues were removed for protein extraction. Western blot analysis proved that β-catenin and Claudin-5 levels were negatively correlated with the protein expression of GRP78 and that PA treatment effectively increased the protein expression levels of β-catenin and Claudin-5 (Figures 7A–D; ^∗^*P* < 0.05; ^∗∗^*P* < 0.01). Western blot analysis also proved that PA treatment decreased the protein expression levels of Bax and C-casp3 (Figures 7A,E–F; ^∗^*P* < 0.05; ^∗∗^*P* < 0.01). Immunofluorescence showed that PA increased connexin levels and restored the function of the BSCB (Figures 7G–I; ^∗∗^*P* < 0.01). Overall, our experimental results showed that, after SCI, the ER stress level increases, the expression of junction proteins is simultaneously reduced, and the expression of apoptosis-related proteins is also increased, causing destruction of the BSCB. PA treatment effectively reverses these consequences.

### PA Inhibits BSCB Rupture and Promotes Functional Recovery After SCI

**FIGURE 8:**
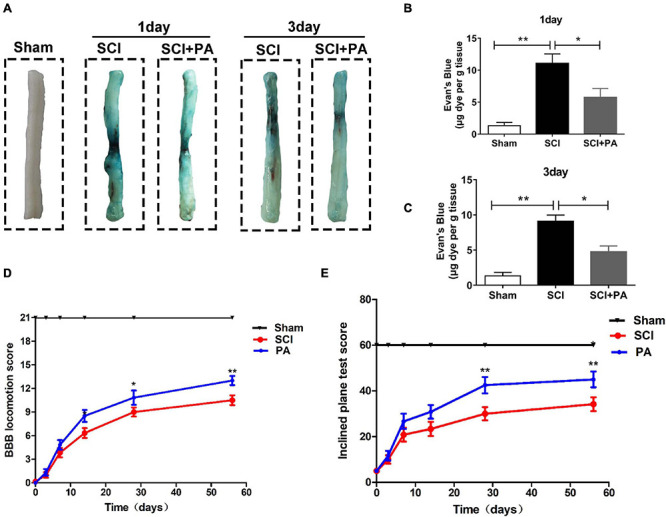
PA inhibits BSCB rupture and promotes functional recovery after SCI. **(A)** As indicated, spinal cords were obtained from the sham, SCI, and PA-treated groups at 1 day **(A)** and 3 days after injury, and a notable reduction in the blue area on the spinal cord surface was observed in the PA-treated group compared with that in the SCI group. **(B,C)** Quantification of Evans blue dye extravasation. The dye content was significantly reduced in the PA-treated group compared with that in the SCI group. **(D)** BBB scores of the sham, SCI, and PA treatment groups 0, 3, 7, 14, 28, and 56 days after SCI. **(E)** Locomotor function was assessed by the inclined plane test. The score of the sham group represents the maximum angle achieved by the rats. “**” represents *P* < 0.01 and “*” represents *P* < 0.05. All the experiments were repeated three times. The data are presented as means ± SD; *n* = 8 per group.

Evans blue dye extravasation was assessed to evaluate the impact of PA on the BSCB. Spinal cord tissues from the sham group showed almost no Evans blue penetration, and Evans blue dye extravasation in the SCI group was significantly higher than that in the PA treatment group at 1 and 3 days (Figure 8A). The permeability of the spinal cord to Evans blue in each group was quantitatively analyzed (Figures 8B,C); permeability was obviously increased in the SCI group compared with that in the sham group (^∗∗^*P* < 0.01). Evans blue dye penetration in the PA treatment group was lower than that in the SCI alone group (^∗^*P* < 0.05). The above results suggested that PA treatment can improve the permeability of the BSCB in rats with SCI. The three groups of rats were subjected to behavioral tests 0, 3, 7, 14, 28, and 56 days after modeling. The BBB locomotor scale (Figure 8D) and inclined plane test (Figure 8E) were performed to confirm that PA promoted functional locomotor recovery after SCI. At 0th day, the average BBB scores of all the groups were approximately 0, indicating that the rat model of SCI was successfully established. The average score of the PA group was markedly higher than that of the SCI group, suggesting that the locomotor function of the model rats was obviously improved after treatment (28 days, ^∗^*P* < 0.05; 56 days, ^∗∗^*P* < 0.01). The scores of the rats in the SCI group and those in the PA treatment group on the inclined plate test were different at 7 days. The average score and standard deviation were significantly higher in the PA treatment group than in the SCI group at 56 days. Thus, PA effectively improves the locomotor ability of rats with SCI.

## Discussion

Spinal cord injury is often accompanied by excitotoxicity and oxidative damage followed by BSCB dysfunction, microvascular injury, and inflammation (Ahuja et al., 2017b). The BSCB is crucial for maintaining homeostasis because it protects neurons and glial cells in the spinal cord (Zhou et al., 2016b; Zheng et al., 2017). The destruction of the BSCB after SCI may result in the production of various harmful bioactive factors, such as TNF-α, inflammatory cytokines and ROS (Kumar et al., 2017; Park et al., 2019). Therefore, maintaining the integrity of the BSCB may be a new strategy to hinder SCI progression.

As a type of naturally tricyclic sesquiterpene, PA exerts protective effects in multiple physiological processes, such as the suppression of inflammation. Previous studies have shown that PA has neuroprotective activities (Wei et al., 2018). However, the effects of PA in SCI remain unknown. According to our studies, treatment with PA effectively reduced the permeability of the BSCB and improved locomotor function in rats. Therefore, we hypothesized that PA might provide beneficial effects on the maintenance of the BSCB. This study may be the first to use PA to treat SCI and identify that PA has protective effects in maintaining BSCB integrity post-SCI.

Next, we sought to identify the mechanism underlying the effect of PA in SCI. We confirmed that PA significantly inhibited the expression of ER stress-related proteins, including CHOP and GRP78, *in vitro* and *in vivo*. CHOP is proapoptotic in cell apoptosis and increases rapidly under ER stress (Guan et al., 2019; Yang et al., 2019b). As a molecular chaperone, GRP78 regulates protein folding in the ER (Ganta and Chaubey, 2019) is suggested to be antiapoptotic during ER stress (Yang et al., 2018; Ardic et al., 2019). Interestingly, GRP78 expression was inhibited in our study. Therefore, we believe that PA may inhibit ER stress upstream of GRP78.

To detect the mechanisms underlying the effect of ER stress on BSCB disruption after SCI, we used TG to increase ER stress. Excessive ER stress had significant effects on decreasing the expression of β-Catenin, ZO-1, and Claudin-5, which are considered vital AJ and TJ proteins (Kim and Kim, 2017); PA can reverse these effects. Our current findings show that ER stress is crucial for BSCB disruption after SCI and PA effectively inhibit BSCB rupture by regulating ER stress.

Furthermore, mitochondrial dysfunction is closely linked to ER stress (Ganta and Chaubey, 2019). CHOP is proapoptotic, and Bcl-2 family proteins mediate the intrinsic apoptotic pathway, leading to caspase-3 activation (Chen et al., 2019). Previous studies have reported that mitochondrial apoptosis is controlled by Bcl-2 family proteins (Sinha et al., 2013). Our studies also proved that PA inhibited the expression of Bax and c-caspase3 and activated Bcl-2. We used JC-1 to evaluate mitochondrial destruction and confirmed that PA decreased mitochondrial death. Taken together, our data suggest that ER stress regulates subsequent mitochondrial destruction and cell apoptosis mediated by mitochondria.

To decipher the role of PA in BSCB maintenance, we performed further experiments. PI3K/AKT acts upstream of ER stress, and AKT is involved in regulating cell apoptosis (Hsu et al., 2017). Activation of the PI3K/AKT pathway attenuates ER stress (Hsu et al., 2017). Our results show that PA treatment induces AKT phosphorylation. Thus, we hypothesized that PA exerts antioxidant effects through the PI3K/AKT pathway. To determine whether PA inhibits ER stress through the PI3K/AKT pathway, LY294002, an effective PI3K inhibitor, was used to inhibit AKT phosphorylation. LY294002 inhibited AKT activation and induced the expression of ER stress-related proteins, and PA did not reverse it. Therefore, PA suppressed ER stress and subsequent BSCB disruption post-SCI by promoting AKT phosphorylation. However, in our results, the phosphorylation of AKT slightly increased. We speculate that this finding may be due to feedback caused by ER stress; thus, further investigations are required to verify this hypothesis in the future.

In conclusion, PA effectively decreases BSCB permeability and promotes functional recovery after SCI. This report is the first to show that PA has a protective effect on the BSCB and that its underlying mechanism is linked to the suppression of ER stress and subsequent mitochondrial dysfunction. PA may be a potential drug to treat central nervous system (CNS) disorders accompanied by BSCB dysfunction. However, our study did not clearly elucidate some questions. We will focus our attention on the specific mechanisms in the future. Taken together, our results suggest that based on its effects and mechanisms, PA may contribute to SCI treatment.

## Data Availability Statement

The original contributions presented in the study are included in the article/supplementary material, further inquiries can be directed to the corresponding authors.

## Ethics Statement

The animal study was reviewed and approved by Wenzhou Medical University.

## Author Contributions

CH, WZ, HJ, and QW designed the experiments. HQ, FQ, YW, and CD performed the vitro the experiments. JZ, MY, ZL, and HQ performed the vivo experiments. WZ, FC, and CH wrote the manuscript. XW, QW, HJ, and HQ revised the manuscript. All authors read and approved the final manuscript.

## Conflict of Interest

The authors declare that the research was conducted in the absence of any commercial or financial relationships that could be construed as a potential conflict of interest.
